# (*S*)-Methyl 2-[(3*R*,4*R*)-2-benzyl-3-(2-fur­yl)-1-oxo-1,2,3,4-tetra­hydro­isoquinoline-4-carboxamido]-3-(1*H*-indol-3-yl)propanoate

**DOI:** 10.1107/S160053680902025X

**Published:** 2009-06-06

**Authors:** Zeliha Baktır, Mehmet Akkurt, Meglena I. Kandinska, Milen G. Bogdanov, Orhan Büyükgüngör

**Affiliations:** a Department of Physics, Faculty of Arts and Sciences, Erciyes University, 38039 Kayseri, Turkey; b Faculty of Chemistry, University of Sofia, 1 James Bourchier blv., 1164 Sofia, Bulgaria; c Department of Physics, Faculty of Arts and Sciences, Ondokuz Mayıs University, 55139 Samsun, Turkey

## Abstract

The title compound, C_33_H_29_N_3_O_5_, was synthesized by the reaction of racemic *trans*-2-benzyl-3-(2-fur­yl)-1-oxo-1,2,3,4-tetra­hydro­isoquinoline-4-carboxylic acid, l-tryptophan methyl ester and diisopropylcarbodiimide in dry dichloro­methane. The furan ring is disordered over two positions in a 0.859 (14):0.141 (14) ratio. In the 1,2,3,4-tetra­hydro­iso­quin­oline ring system, the heterocyclic ring is not planar, with puckering parameters *Q*_T_ = 0.448 (2) Å, θ = 64.9 (3) and ϕ = 268.3 (3)°. The crystal is extended into a three-dimensional supra­molecular architecture through inter­molecular N—H⋯O hydrogen bonds and C—H⋯π inter­actions. The absolute structure was assigned by reference to the chiral starting material.

## Related literature

For the synthesis of new heterocyclic compounds with pharmacological activities, see: Bogdanov *et al.* (2007 ▶); Burdzhiev & Stanoeva (2006 ▶); Kandinska *et al.* (2006 ▶). For ring conformation analysis, see: Cremer & Pople (1975 ▶).
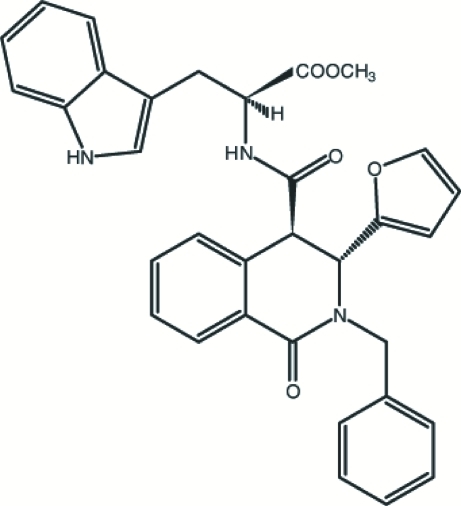


## Experimental

### 

#### Crystal data

C_33_H_29_N_3_O_5_
*M* *_r_* = 547.59
Monoclinic, 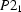

*a* = 8.6866 (5) Å
*b* = 15.8630 (7) Å
*c* = 10.5480 (6) Å
β = 104.543 (5)°
*V* = 1406.90 (13) Å^3^
*Z* = 2
Mo *K*α radiation
μ = 0.09 mm^−1^
*T* = 296 K
0.60 × 0.54 × 0.36 mm

#### Data collection

Stoe IPDS-2 diffractometer
Absorption correction: integration (*X-RED32*; Stoe & Cie, 2002 ▶) *T*_min_ = 0.949, *T*_max_ = 0.969
8814 measured reflections
3010 independent reflections
2613 reflections with *I* > 2σ(*I*)
*R*_int_ = 0.034

#### Refinement

*R*[*F* ^2^ > 2σ(*F* ^2^)] = 0.033
*wR*(*F* ^2^) = 0.082
*S* = 1.02
3010 reflections
409 parameters
16 restraints
H-atom parameters constrained
Δρ_max_ = 0.11 e Å^−3^
Δρ_min_ = −0.10 e Å^−3^

### 

Data collection: *X-AREA* (Stoe & Cie, 2002 ▶); cell refinement: *X-AREA*; data reduction: *X-RED32* (Stoe & Cie, 2002 ▶); program(s) used to solve structure: *SIR97* (Altomare *et al.*, 1999 ▶); program(s) used to refine structure: *SHELXL97* (Sheldrick, 2008 ▶); molecular graphics: *ORTEP-3* (Farrugia, 1997 ▶); software used to prepare material for publication: *WinGX* (Farrugia, 1999 ▶).

## Supplementary Material

Crystal structure: contains datablocks global, I. DOI: 10.1107/S160053680902025X/hb2987sup1.cif

Structure factors: contains datablocks I. DOI: 10.1107/S160053680902025X/hb2987Isup2.hkl

Additional supplementary materials:  crystallographic information; 3D view; checkCIF report

## Figures and Tables

**Table 1:** Hydrogen-bond geometry (Å, °)

*D*—H⋯*A*	*D*—H	H⋯*A*	*D*⋯*A*	*D*—H⋯*A*
N1—H1⋯O3^i^	0.86	2.12	2.942 (3)	161
C12—H12*A*⋯*Cg*5^ii^	0.96	2.63	3.551 (5)	160

Symmetry codes: (i) 
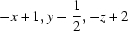
; (ii) 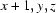. *Cg*5 is the centroid of the C1–C6 ring.

**Fig. 1.**
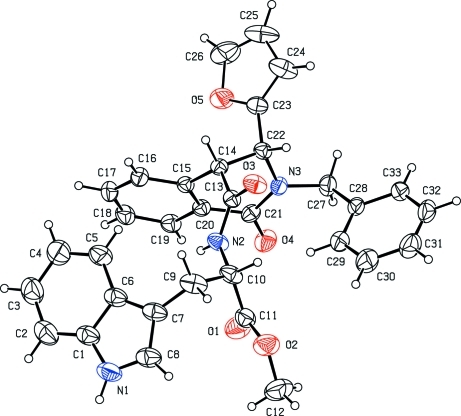
View of the title molecule (I) with displacement ellipsoids drawn at the 30% probability level. The minor component of the disordered furan ring has been omitted, for clarity.

**Fig. 2.**
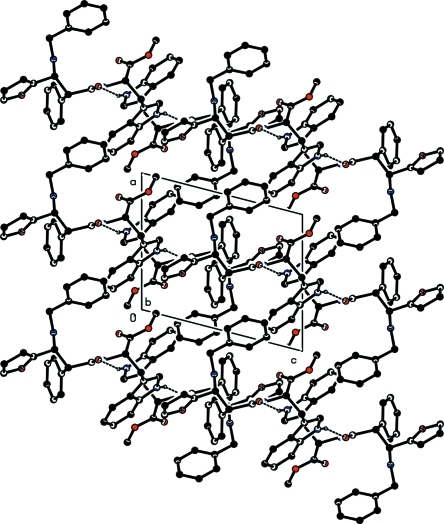
The hydrogen bondging of (I) showing that molecules linked through intermolecular N–H···O bonding. For the sake of clarity, the H-atoms not involved in H-bonding and the minor component of the disordered furan ring have been omitted.

## supplementary crystallographic information

### Comment

As a part of our studies on the synthesis of new heterocyclic compounds with expected pharmacological activities carried out in our laboratory (Bogdanov *et al.*, 2007; Burdzhiev & Stanoeva, 2006; Kandinska *et al.*, 2006) we focused our attention to some 3,4-dihydroisoquinolin-1-ones, containing amino acid moieties. The title compound (I) was synthesized while searching for new ACE inhibitors of this type. The structure of (I) was determined by spectral analysis (^1^HNMR & IR) and microanalysis. In this paper, we report the X-ray crystallographic studies of (I).

In the title molecule, (Fig. 1), the furan ring is of positional disorder, with a 0.859 (14):0.141 (14) ratio of site occupancy. There is a dihedral angle of 28.7 (12) ° between the planes of the major and minor components (O5/C23—C26) and (O5'/C23/C24'-C26') of the disordered furan ring. In the 1,2,3,4-tetrahydroisoquinoline ring system (N3/C14—C22), the six-membered ring (N3/C14/C15/C20—C22) is not planar with the puckering parameters of Q_T_ = 0.448 (2) Å, θ = 64.9 (3) ° and φ = 268.3 (3) ° (Cremer & Pople, 1975). In the 1*H*-indole ring system (N1/C1—C8), the dihedral angle between the planes of the six- and five-membered rings is 2.60 (17)°. The values of the dihedral angles between the other ring planes [A(O5/C23—C26), A'(O5'/C23/C24'-C26'), B(C15—C20), C(C28—C33), D(C1—C6) and E(N1/C1/C6—C8)] are: A/B = 78.5 (3) °, A'/B = 66.2 (11) °, A/C = 25.4 (3) °, A'/C = 50.9 (11) °, A/D = 9.5 (3) °, A'/D = 30.7 (11) °, A/E = 7.2 (3) °, A'/E = 29.0 (11) °, B/C = 78.13 (12) °, B/D = 70.54 (14) °, B/E = 71.98 (14) °, C/D = 31.63 (16) ° and C/E = 30.60 (16)°.

The crystal is extended into three dimensional supramolecular architecture through intermolecular N—H···O hydrogen bonds (Fig. 2) and C—H···π interactions (Table 1).

### Experimental

Compound (I) was synthesized by the reaction of racemic *trans*-2-benzyl-3-(furan-2-yl)-1-oxo-1,2,3,4-tetrahydroisoquinoline-4carboxylic acid (Kandinska *et al.*, 2006) (0.347 g, 0.001 mol), *L*-tryptophan methylester (0.217 g, 0.001 mol) and diisopropilcarbodiimide (0.20 ml, 0.0013 mol) in dry dichloromethane (3 ml). The reaction mixture was stirred at 263 K for 1 h. Resulting diisopropylurea was filtered and washed with dichloromethane. After evaporation of the solvent, the crude product was dissolved in ethyl acetate and washed once with HCl (1:1), once with 10% sodium carbonate and three times with water. The organic layer was dried (sodium sulfate) and evaporated to dryness. Resulting oil (yield 93%, 0.508 g, mixture of diastereomers) was purified by column chromatography with hexane/ethyl acetate (3:2) (yield 88%, 0.480 g, mixture of diastereomers) and compound (I) with smaller *R*_F_ was isolated as asingle diastereomer (m.p. 422–424 K). Single crystals of (I) were obtained by slow evaporation from hexane/ethyl acetate solution (3:2) at room temperature. Analysis, calculated for C_33_H_29_N_3_O_5_: C 72.38, H 5.34, N 7.67 (%); found C 72.77, H 5.73, N 7.66 (%). IR (chloroform) 1640, 1660 cm^-1^ (C=O, amide), 1730 cm^-1^ (C=O, ester), 3440 cm^-1 ^(NH, Trp). The ^1^H NMR spectra of (I) were obtained on a Bruker DRX250 spectrometer at 250.13 MHz in CDCl_3_ at 293 K. Chemical shifts δ are expressed in parts per million (p.p.m.) from tetramethylsilane as an internal standard. ^1^H NMR (250 MHz, CDCl_3_, p.p.m.): δ = 3.12 (dd, 1H, H*^a^*—CH_2_—Trp, J = 5.0; 15.0 Hz); 3.24 (dd, 1H, H*^b^*—CH_2_—Trp, J = 5.0; 15.0 Hz); 3.58 (s, 3H, H-COOCH_3_); 3.82 (d, 1H, H-4, J = 1.8 Hz); 4.13 (d, 1H, *H^a^*-benzyl, J = 14.5 H); 4.63 (dt, 1H, H—CH-Trp, J = 5.0; 7.8 Hz); 5.29 (d, 1H, H*^b^*– benzyl, J = 14.5 Hz); 5.37 (d, 1H, H-3, J = 1.8 Hz); 5.57 (d, 1H, NH-amide, J = 7.8 Hz); 5.80 (d, 1H, H-10, J = 3.3 Hz); 6.09 (dd, 1H, H-9, J = 2.0; 3.3 Hz); 6.60 (d, 1H, H-Ind, J = 7.3 Hz); 6.64 (d, 1H, H-Ind, J = 2.3 Hz); 7.07–7.19 (m, 4H, H-Ind, H-5, 2*H*-Ph); 7.21–7.24 (m, 5H, H-11, H-Ind, 3*H*-Ph); 7.28–7.35 (m, 2H, H-6,7); 7.41 (d, 1H, H-Ind, J = 8.3 Hz); 8.08 (bs, 1H, NH-Ind); 8.14 (d, 1H, H-8, J = 1.3; 7.8 Hz).

### Refinement

Anomalous dispersion was negligible and Friedel pairs were merged before refinement. H-atoms were positioned geometrically, with N—H = 0.86 Å and C—H = 0.93–0.98 Å and refined as riding with *U*_iso_(H) = 1.2 or 1.5*U*_eq_(carrier). The refined site occupancies for the major and minor components of the disordered furan ring has a ratio of 0.859 (14):0.141 (14). Geometrical restraints were applied to the positional parameters of the disordered atoms.

### Crystal data

**Table:**

C_33_H_29_N_3_O_5_	*F*(000) = 576
*M**_r_* = 547.59	*D*_x_ = 1.293 Mg m^−^^3^
Monoclinic, *P*2_1_	Mo *K*α radiation, λ = 0.71073 Å
Hall symbol: P 2yb	Cell parameters from 15289 reflections
*a* = 8.6866 (5) Å	θ = 2.0–28.1°
*b* = 15.8630 (7) Å	µ = 0.09 mm^−^^1^
*c* = 10.5480 (6) Å	*T* = 296 K
β = 104.543 (5)°	Prism, colourless
*V* = 1406.90 (13) Å^3^	0.60 × 0.54 × 0.36 mm
*Z* = 2	

### Data collection

**Table:**

Stoe IPDS-2 diffractometer	3010 independent reflections
Radiation source: sealed X-ray tube, 12 x 0.4 mm long-fine focus	2613 reflections with *I* > 2σ(*I*)
plane graphite	*R*_int_ = 0.034
Detector resolution: 6.67 pixels mm^-1^	θ_max_ = 26.5°, θ_min_ = 2.0°
ω scans	*h* = −10→10
Absorption correction: integration (*X-RED32*; Stoe & Cie, 2002)	*k* = −19→19
*T*_min_ = 0.949, *T*_max_ = 0.969	*l* = −13→13
8814 measured reflections	

### Refinement

**Table:**

Refinement on *F*^2^	Secondary atom site location: difference Fourier map
Least-squares matrix: full	Hydrogen site location: inferred from neighbouring sites
*R*[*F*^2^ > 2σ(*F*^2^)] = 0.033	H-atom parameters constrained
*wR*(*F*^2^) = 0.082	*w* = 1/[σ^2^(*F*_o_^2^) + (0.0477*P*)^2^ + 0.0571*P*] where *P* = (*F*_o_^2^ + 2*F*_c_^2^)/3
*S* = 1.02	(Δ/σ)_max_ < 0.001
3010 reflections	Δρ_max_ = 0.11 e Å^−^^3^
409 parameters	Δρ_min_ = −0.10 e Å^−^^3^
16 restraints	Extinction correction: *SHELXL97* (Sheldrick, 2008), Fc^*^=kFc[1+0.001Fc^2^λ^3^/sin(2θ)]^-1/4^
Primary atom site location: structure-invariant direct methods	Extinction coefficient: 0.015 (2)

### Special details

**Table:**

Geometry. Bond distances, angles *etc*. have been calculated using the rounded fractional coordinates. All su's are estimated from the variances of the (full) variance-covariance matrix. The cell e.s.d.'s are taken into account in the estimation of distances, angles and torsion angles
Refinement. Refinement on *F*^2^ for ALL reflections except those flagged by the user for potential systematic errors. Weighted *R*-factors *wR* and all goodnesses of fit *S* are based on *F*^2^, conventional *R*-factors *R* are based on *F*, with *F* set to zero for negative *F*^2^. The observed criterion of *F*^2^ > σ(*F*^2^) is used only for calculating -*R*-factor-obs *etc*. and is not relevant to the choice of reflections for refinement. *R*-factors based on *F*^2^ are statistically about twice as large as those based on *F*, and *R*-factors based on ALL data will be even larger.

### Fractional atomic coordinates and isotropic or equivalent isotropic displacement parameters (Å^2^)

**Table:**

	*x*	*y*	*z*	*U*_iso_*/*U*_eq_	Occ. (<1)
O1	0.7796 (3)	0.13450 (15)	0.8595 (2)	0.0877 (8)	
O2	0.8751 (3)	0.20882 (15)	1.04255 (18)	0.0900 (8)	
O3	0.5503 (2)	0.39216 (9)	0.73029 (15)	0.0623 (5)	
O4	0.7409 (2)	0.14390 (12)	0.4350 (2)	0.0714 (7)	
O5	0.3408 (6)	0.3203 (3)	0.2533 (3)	0.0832 (15)	0.859 (14)
N1	0.4577 (3)	0.03542 (17)	1.0943 (2)	0.0817 (10)	
N2	0.5562 (3)	0.25261 (12)	0.76349 (16)	0.0559 (6)	
N3	0.6645 (2)	0.27926 (11)	0.45137 (17)	0.0475 (5)	
C1	0.3339 (4)	0.04232 (19)	0.9851 (3)	0.0717 (10)	
C2	0.2120 (5)	−0.0141 (2)	0.9326 (3)	0.0926 (14)	
C3	0.0984 (5)	0.0105 (3)	0.8234 (3)	0.0994 (14)	
C4	0.1055 (5)	0.0888 (3)	0.7664 (3)	0.1012 (13)	
C5	0.2279 (4)	0.1442 (2)	0.8179 (3)	0.0822 (11)	
C6	0.3448 (3)	0.12196 (17)	0.9300 (2)	0.0655 (9)	
C7	0.4798 (3)	0.16422 (17)	1.0120 (2)	0.0654 (9)	
C8	0.5422 (4)	0.1086 (2)	1.1101 (2)	0.0751 (10)	
C9	0.5382 (4)	0.25128 (18)	0.9959 (2)	0.0731 (9)	
C10	0.6440 (3)	0.25915 (15)	0.8994 (2)	0.0631 (8)	
C11	0.7722 (3)	0.19342 (17)	0.9276 (2)	0.0638 (8)	
C12	0.9983 (5)	0.1470 (3)	1.0888 (4)	0.1111 (14)	
C13	0.5186 (2)	0.32068 (13)	0.68794 (18)	0.0442 (6)	
C14	0.4349 (2)	0.30917 (13)	0.54335 (18)	0.0424 (6)	
C15	0.3774 (2)	0.22153 (12)	0.50037 (18)	0.0422 (6)	
C16	0.2255 (3)	0.19519 (15)	0.4982 (2)	0.0525 (7)	
C17	0.1728 (3)	0.11632 (16)	0.4523 (2)	0.0596 (8)	
C18	0.2738 (3)	0.06173 (15)	0.4106 (2)	0.0598 (8)	
C19	0.4266 (3)	0.08634 (14)	0.4148 (2)	0.0541 (7)	
C20	0.4788 (2)	0.16665 (13)	0.45772 (18)	0.0449 (6)	
C21	0.6383 (2)	0.19472 (14)	0.44794 (19)	0.0490 (7)	
C22	0.5424 (2)	0.34225 (13)	0.45839 (18)	0.0457 (6)	
C23	0.4504 (3)	0.37158 (14)	0.3274 (2)	0.0552 (7)	
C24	0.4590 (11)	0.4436 (3)	0.2647 (4)	0.0905 (19)	0.859 (14)
C25	0.3411 (11)	0.4410 (4)	0.1445 (5)	0.101 (2)	0.859 (14)
C26	0.2724 (10)	0.3674 (7)	0.1422 (7)	0.106 (3)	0.859 (14)
C27	0.8112 (3)	0.31240 (18)	0.4263 (2)	0.0591 (8)	
C28	0.9227 (2)	0.34976 (14)	0.5463 (2)	0.0502 (7)	
C29	0.9300 (3)	0.3188 (2)	0.6691 (2)	0.0682 (8)	
C30	1.0360 (4)	0.3530 (3)	0.7775 (3)	0.0895 (13)	
C31	1.1350 (4)	0.4169 (2)	0.7639 (3)	0.0872 (11)	
C32	1.1294 (3)	0.44850 (17)	0.6434 (3)	0.0751 (9)	
C33	1.0234 (3)	0.41541 (15)	0.5341 (3)	0.0615 (8)	
C25'	0.273 (4)	0.346 (2)	0.134 (3)	0.069 (10)	0.141 (14)
C26'	0.280 (3)	0.4291 (17)	0.162 (3)	0.088 (13)	0.141 (14)
C24'	0.385 (3)	0.3089 (12)	0.246 (2)	0.043 (6)	0.141 (14)
O5'	0.390 (3)	0.4486 (14)	0.281 (3)	0.127 (13)	0.141 (14)
H3	0.01520	−0.02590	0.78690	0.1190*	
H2A	0.52760	0.20370	0.73080	0.0670*	
H8	0.63080	0.11920	1.17880	0.0900*	
H4	0.02690	0.10420	0.69260	0.1210*	
H5	0.23240	0.19610	0.77810	0.0990*	
H10	0.69520	0.31460	0.91210	0.0760*	
H12A	1.05840	0.13960	1.02470	0.1340*	
H12B	0.95090	0.09430	1.10290	0.1340*	
H12C	1.06760	0.16600	1.16960	0.1340*	
H14	0.34070	0.34550	0.52550	0.0510*	
H9A	0.59770	0.27150	1.08090	0.0880*	
H9B	0.44690	0.28800	0.96650	0.0880*	
H18	0.23840	0.00840	0.37970	0.0720*	
H19	0.49550	0.04910	0.38890	0.0650*	
H22	0.59860	0.39150	0.50370	0.0550*	
H24	0.52950	0.48760	0.29450	0.1090*	0.859 (14)
H25	0.31740	0.48270	0.08070	0.1220*	0.859 (14)
H26	0.18840	0.34880	0.07470	0.1270*	0.859 (14)
H27A	0.78420	0.35540	0.35890	0.0710*	
H27B	0.86550	0.26720	0.39320	0.0710*	
H29	0.86360	0.27480	0.67960	0.0820*	
H30	1.03940	0.33200	0.86040	0.1070*	
H31	1.20660	0.43880	0.83730	0.1050*	
H32	1.19670	0.49240	0.63410	0.0900*	
H33	1.01980	0.43740	0.45170	0.0740*	
H16	0.15770	0.23110	0.52810	0.0630*	
H17	0.06920	0.09980	0.44940	0.0720*	
H1	0.47870	−0.00800	1.14460	0.0980*	
H2	0.20770	−0.06670	0.97030	0.1110*	
H24'	0.40720	0.25180	0.25950	0.0520*	0.141 (14)
H25'	0.21150	0.31920	0.06070	0.0840*	0.141 (14)
H26'	0.21840	0.46940	0.10880	0.1060*	0.141 (14)

### Atomic displacement parameters (Å^2^)

**Table:**

	*U*^11^	*U*^22^	*U*^33^	*U*^12^	*U*^13^	*U*^23^
O1	0.0850 (14)	0.0796 (14)	0.0907 (14)	0.0121 (11)	0.0078 (11)	−0.0070 (11)
O2	0.0954 (15)	0.0963 (16)	0.0643 (11)	−0.0005 (12)	−0.0062 (10)	0.0093 (10)
O3	0.0906 (12)	0.0369 (8)	0.0542 (8)	−0.0009 (8)	0.0084 (8)	−0.0075 (7)
O4	0.0567 (10)	0.0609 (11)	0.0991 (13)	0.0151 (9)	0.0240 (9)	−0.0105 (9)
O5	0.086 (3)	0.092 (3)	0.0578 (17)	−0.009 (2)	−0.0076 (14)	0.0135 (15)
N1	0.0965 (18)	0.0772 (17)	0.0752 (15)	0.0155 (15)	0.0288 (13)	0.0313 (12)
N2	0.0781 (13)	0.0367 (9)	0.0477 (9)	−0.0064 (9)	0.0062 (8)	−0.0007 (7)
N3	0.0401 (8)	0.0474 (10)	0.0548 (9)	−0.0010 (8)	0.0116 (7)	−0.0024 (7)
C1	0.0840 (19)	0.0725 (17)	0.0662 (14)	0.0151 (15)	0.0333 (14)	0.0193 (13)
C2	0.111 (3)	0.083 (2)	0.095 (2)	−0.003 (2)	0.047 (2)	0.0175 (18)
C3	0.099 (2)	0.119 (3)	0.086 (2)	−0.023 (2)	0.0338 (19)	0.013 (2)
C4	0.091 (2)	0.140 (3)	0.0739 (18)	−0.010 (2)	0.0232 (16)	0.027 (2)
C5	0.088 (2)	0.094 (2)	0.0674 (16)	0.0045 (18)	0.0250 (15)	0.0286 (15)
C6	0.0792 (16)	0.0700 (16)	0.0543 (12)	0.0158 (14)	0.0301 (12)	0.0154 (11)
C7	0.0897 (18)	0.0642 (15)	0.0479 (12)	0.0188 (14)	0.0277 (12)	0.0099 (11)
C8	0.0902 (18)	0.084 (2)	0.0525 (13)	0.0158 (17)	0.0207 (12)	0.0141 (13)
C9	0.110 (2)	0.0568 (14)	0.0521 (12)	0.0172 (15)	0.0195 (13)	−0.0027 (11)
C10	0.0926 (18)	0.0425 (11)	0.0479 (11)	−0.0084 (12)	0.0058 (11)	0.0001 (9)
C11	0.0714 (15)	0.0610 (15)	0.0554 (12)	−0.0085 (13)	0.0095 (11)	0.0075 (11)
C12	0.092 (2)	0.141 (3)	0.086 (2)	0.018 (2)	−0.0044 (18)	0.030 (2)
C13	0.0492 (10)	0.0376 (10)	0.0473 (10)	0.0005 (9)	0.0149 (8)	−0.0032 (8)
C14	0.0434 (9)	0.0355 (10)	0.0479 (10)	0.0042 (8)	0.0105 (8)	−0.0021 (8)
C15	0.0445 (10)	0.0378 (10)	0.0423 (9)	−0.0006 (8)	0.0074 (8)	0.0002 (7)
C16	0.0470 (11)	0.0539 (13)	0.0571 (12)	−0.0009 (11)	0.0138 (9)	−0.0017 (10)
C17	0.0518 (12)	0.0590 (14)	0.0651 (13)	−0.0142 (11)	0.0092 (10)	0.0024 (11)
C18	0.0699 (15)	0.0418 (12)	0.0622 (13)	−0.0121 (11)	0.0064 (11)	−0.0043 (10)
C19	0.0627 (13)	0.0405 (12)	0.0578 (12)	0.0037 (10)	0.0128 (10)	−0.0044 (9)
C20	0.0475 (11)	0.0379 (10)	0.0474 (10)	0.0021 (9)	0.0083 (8)	−0.0024 (8)
C21	0.0451 (11)	0.0486 (12)	0.0515 (11)	0.0078 (10)	0.0088 (9)	−0.0064 (9)
C22	0.0515 (11)	0.0375 (10)	0.0464 (10)	−0.0007 (9)	0.0094 (8)	−0.0005 (8)
C23	0.0657 (14)	0.0493 (13)	0.0505 (11)	0.0076 (11)	0.0144 (10)	0.0010 (9)
C24	0.140 (5)	0.058 (2)	0.067 (2)	0.001 (3)	0.014 (3)	0.0133 (17)
C25	0.156 (6)	0.089 (3)	0.059 (2)	0.053 (4)	0.027 (3)	0.025 (2)
C26	0.105 (6)	0.142 (7)	0.054 (3)	0.011 (5)	−0.014 (3)	0.009 (4)
C27	0.0488 (11)	0.0696 (15)	0.0609 (13)	−0.0075 (12)	0.0175 (10)	0.0001 (11)
C28	0.0380 (10)	0.0452 (11)	0.0675 (13)	0.0024 (9)	0.0133 (9)	0.0017 (9)
C29	0.0559 (13)	0.0757 (16)	0.0675 (14)	−0.0098 (13)	0.0052 (11)	0.0103 (13)
C30	0.0757 (17)	0.113 (3)	0.0682 (16)	−0.0181 (18)	−0.0037 (13)	0.0065 (16)
C31	0.0659 (17)	0.093 (2)	0.090 (2)	−0.0139 (17)	−0.0041 (14)	−0.0100 (17)
C32	0.0493 (13)	0.0509 (14)	0.121 (2)	−0.0071 (12)	0.0136 (14)	−0.0062 (15)
C33	0.0497 (12)	0.0516 (13)	0.0825 (16)	0.0002 (11)	0.0152 (11)	0.0099 (11)
C25'	0.09 (2)	0.077 (17)	0.052 (16)	0.047 (16)	0.039 (16)	0.031 (12)
C26'	0.037 (11)	0.15 (3)	0.08 (2)	0.004 (15)	0.019 (11)	0.08 (2)
C24'	0.045 (11)	0.032 (9)	0.051 (10)	0.007 (8)	0.010 (8)	−0.010 (7)
O5'	0.12 (2)	0.088 (15)	0.18 (3)	0.059 (14)	0.049 (17)	0.023 (14)

### Geometric parameters (Å, °)

**Table:**

O1—C11	1.190 (3)	C24—C25	1.416 (9)
O2—C11	1.336 (3)	C24'—C25'	1.45 (4)
O2—C12	1.443 (5)	C25—C26	1.309 (13)
O3—C13	1.225 (2)	C25'—C26'	1.35 (4)
O4—C21	1.235 (3)	C27—C28	1.509 (3)
O5—C23	1.344 (5)	C28—C33	1.387 (3)
O5—C26	1.391 (9)	C28—C29	1.372 (3)
O5'—C26'	1.41 (4)	C29—C30	1.386 (4)
O5'—C23	1.37 (2)	C30—C31	1.360 (5)
N1—C8	1.361 (4)	C31—C32	1.356 (4)
N1—C1	1.369 (4)	C32—C33	1.385 (4)
N2—C13	1.333 (3)	C2—H2	0.9300
N2—C10	1.449 (3)	C3—H3	0.9300
N3—C22	1.473 (3)	C4—H4	0.9300
N3—C21	1.359 (3)	C5—H5	0.9300
N3—C27	1.463 (3)	C8—H8	0.9300
N1—H1	0.8600	C9—H9A	0.9700
N2—H2A	0.8600	C9—H9B	0.9700
C1—C6	1.404 (4)	C10—H10	0.9800
C1—C2	1.391 (5)	C12—H12A	0.9600
C2—C3	1.372 (5)	C12—H12B	0.9600
C3—C4	1.388 (6)	C12—H12C	0.9600
C4—C5	1.381 (6)	C14—H14	0.9800
C5—C6	1.397 (4)	C16—H16	0.9300
C6—C7	1.436 (4)	C17—H17	0.9300
C7—C8	1.364 (4)	C18—H18	0.9300
C7—C9	1.496 (4)	C19—H19	0.9300
C9—C10	1.538 (4)	C22—H22	0.9800
C10—C11	1.500 (4)	C24—H24	0.9300
C13—C14	1.527 (3)	C24'—H24'	0.9300
C14—C22	1.540 (3)	C25—H25	0.9300
C14—C15	1.508 (3)	C25'—H25'	0.9300
C15—C16	1.379 (3)	C26—H26	0.9300
C15—C20	1.391 (3)	C26'—H26'	0.9300
C16—C17	1.378 (3)	C27—H27A	0.9700
C17—C18	1.381 (4)	C27—H27B	0.9700
C18—C19	1.374 (4)	C29—H29	0.9300
C19—C20	1.389 (3)	C30—H30	0.9300
C20—C21	1.483 (3)	C31—H31	0.9300
C22—C23	1.487 (3)	C32—H32	0.9300
C23—C24'	1.34 (2)	C33—H33	0.9300
C23—C24	1.332 (5)		
			
C11—O2—C12	116.9 (3)	C28—C29—C30	120.2 (3)
C23—O5—C26	104.8 (5)	C29—C30—C31	120.8 (3)
C23—O5'—C26'	103.6 (19)	C30—C31—C32	120.0 (3)
C1—N1—C8	108.7 (2)	C31—C32—C33	120.0 (3)
C10—N2—C13	121.51 (19)	C28—C33—C32	120.7 (3)
C21—N3—C22	123.57 (17)	C1—C2—H2	121.00
C22—N3—C27	115.88 (18)	C3—C2—H2	121.00
C21—N3—C27	119.83 (18)	C2—C3—H3	119.00
C1—N1—H1	126.00	C4—C3—H3	119.00
C8—N1—H1	126.00	C3—C4—H4	120.00
C10—N2—H2A	119.00	C5—C4—H4	120.00
C13—N2—H2A	119.00	C4—C5—H5	120.00
N1—C1—C6	107.5 (3)	C6—C5—H5	120.00
C2—C1—C6	122.6 (3)	N1—C8—H8	124.00
N1—C1—C2	130.0 (3)	C7—C8—H8	125.00
C1—C2—C3	117.8 (3)	C7—C9—H9A	109.00
C2—C3—C4	121.2 (4)	C7—C9—H9B	109.00
C3—C4—C5	120.8 (3)	C10—C9—H9A	108.00
C4—C5—C6	119.8 (3)	C10—C9—H9B	108.00
C5—C6—C7	134.6 (3)	H9A—C9—H9B	107.00
C1—C6—C5	117.9 (3)	N2—C10—H10	108.00
C1—C6—C7	107.5 (2)	C9—C10—H10	108.00
C6—C7—C9	127.7 (2)	C11—C10—H10	108.00
C6—C7—C8	105.3 (2)	O2—C12—H12A	109.00
C8—C7—C9	127.0 (2)	O2—C12—H12B	109.00
N1—C8—C7	111.1 (2)	O2—C12—H12C	109.00
C7—C9—C10	115.2 (2)	H12A—C12—H12B	109.00
C9—C10—C11	110.5 (2)	H12A—C12—H12C	109.00
N2—C10—C11	109.07 (19)	H12B—C12—H12C	109.00
N2—C10—C9	113.3 (2)	C13—C14—H14	107.00
O1—C11—O2	124.5 (3)	C15—C14—H14	107.00
O1—C11—C10	125.2 (2)	C22—C14—H14	107.00
O2—C11—C10	110.3 (2)	C15—C16—H16	120.00
O3—C13—N2	122.22 (18)	C17—C16—H16	120.00
N2—C13—C14	118.95 (18)	C16—C17—H17	120.00
O3—C13—C14	118.83 (18)	C18—C17—H17	120.00
C15—C14—C22	110.04 (15)	C17—C18—H18	120.00
C13—C14—C15	116.70 (16)	C19—C18—H18	120.00
C13—C14—C22	109.53 (15)	C18—C19—H19	120.00
C14—C15—C20	119.04 (16)	C20—C19—H19	120.00
C16—C15—C20	119.08 (18)	N3—C22—H22	107.00
C14—C15—C16	121.85 (18)	C14—C22—H22	107.00
C15—C16—C17	120.9 (2)	C23—C22—H22	107.00
C16—C17—C18	120.0 (2)	C23—C24—H24	126.00
C17—C18—C19	119.8 (2)	C25—C24—H24	126.00
C18—C19—C20	120.3 (2)	C23—C24'—H24'	126.00
C19—C20—C21	119.12 (19)	C25'—C24'—H24'	126.00
C15—C20—C19	119.86 (19)	C24—C25—H25	127.00
C15—C20—C21	120.86 (18)	C26—C25—H25	127.00
O4—C21—C20	121.6 (2)	C24'—C25'—H25'	128.00
N3—C21—C20	116.58 (17)	C26'—C25'—H25'	128.00
O4—C21—N3	121.77 (18)	O5—C26—H26	124.00
C14—C22—C23	112.62 (17)	C25—C26—H26	124.00
N3—C22—C14	110.35 (16)	O5'—C26'—H26'	123.00
N3—C22—C23	113.05 (17)	C25'—C26'—H26'	124.00
O5'—C23—C24'	111.7 (15)	N3—C27—H27B	109.00
C22—C23—C24	130.3 (3)	C28—C27—H27A	109.00
C22—C23—C24'	113.9 (9)	N3—C27—H27A	109.00
O5—C23—C24	110.5 (4)	H27A—C27—H27B	108.00
O5—C23—C22	119.2 (2)	C28—C27—H27B	109.00
O5'—C23—C22	132.8 (12)	C28—C29—H29	120.00
C23—C24—C25	107.6 (5)	C30—C29—H29	120.00
C23—C24'—C25'	108.0 (18)	C29—C30—H30	120.00
C24—C25—C26	105.5 (6)	C31—C30—H30	120.00
C24'—C25'—C26'	104 (2)	C32—C31—H31	120.00
O5—C26—C25	111.6 (7)	C30—C31—H31	120.00
O5'—C26'—C25'	113 (3)	C31—C32—H32	120.00
N3—C27—C28	113.23 (17)	C33—C32—H32	120.00
C27—C28—C33	120.3 (2)	C32—C33—H33	120.00
C29—C28—C33	118.4 (2)	C28—C33—H33	120.00
C27—C28—C29	121.3 (2)		
			
C12—O2—C11—C10	175.0 (3)	N2—C10—C11—O1	−14.8 (4)
C12—O2—C11—O1	−3.2 (4)	N2—C13—C14—C22	−116.6 (2)
C23—O5—C26—C25	3.2 (9)	N2—C13—C14—C15	9.3 (3)
C26—O5—C23—C22	177.2 (4)	O3—C13—C14—C22	62.7 (2)
C26—O5—C23—C24	−4.0 (7)	O3—C13—C14—C15	−171.43 (17)
C8—N1—C1—C6	1.4 (3)	C13—C14—C15—C20	−89.9 (2)
C1—N1—C8—C7	−1.3 (4)	C15—C14—C22—C23	77.8 (2)
C8—N1—C1—C2	−177.0 (4)	C22—C14—C15—C16	−142.20 (18)
C13—N2—C10—C11	−135.0 (2)	C22—C14—C15—C20	35.7 (2)
C10—N2—C13—C14	176.3 (2)	C15—C14—C22—N3	−49.5 (2)
C13—N2—C10—C9	101.6 (3)	C13—C14—C15—C16	92.2 (2)
C10—N2—C13—O3	−3.0 (4)	C13—C14—C22—C23	−152.62 (17)
C27—N3—C21—C20	−172.55 (17)	C13—C14—C22—N3	80.0 (2)
C22—N3—C21—C20	−2.7 (3)	C14—C15—C16—C17	176.68 (19)
C27—N3—C22—C23	78.7 (2)	C20—C15—C16—C17	−1.2 (3)
C21—N3—C22—C23	−91.5 (2)	C16—C15—C20—C19	−0.5 (3)
C21—N3—C22—C14	35.6 (2)	C14—C15—C20—C21	−3.1 (3)
C27—N3—C21—O4	6.2 (3)	C16—C15—C20—C21	174.88 (18)
C27—N3—C22—C14	−154.15 (16)	C14—C15—C20—C19	−178.47 (18)
C22—N3—C27—C28	78.7 (2)	C15—C16—C17—C18	1.6 (3)
C21—N3—C27—C28	−110.7 (2)	C16—C17—C18—C19	−0.1 (3)
C22—N3—C21—O4	176.06 (19)	C17—C18—C19—C20	−1.6 (3)
C2—C1—C6—C7	177.5 (3)	C18—C19—C20—C15	1.9 (3)
C2—C1—C6—C5	−0.2 (5)	C18—C19—C20—C21	−173.54 (19)
N1—C1—C6—C7	−1.0 (3)	C15—C20—C21—O4	165.9 (2)
N1—C1—C6—C5	−178.7 (3)	C19—C20—C21—N3	160.05 (18)
C6—C1—C2—C3	−0.6 (5)	C19—C20—C21—O4	−18.7 (3)
N1—C1—C2—C3	177.5 (4)	C15—C20—C21—N3	−15.4 (3)
C1—C2—C3—C4	0.6 (6)	N3—C22—C23—C24	−104.3 (5)
C2—C3—C4—C5	0.3 (6)	N3—C22—C23—O5	74.3 (3)
C3—C4—C5—C6	−1.1 (6)	C14—C22—C23—C24	129.8 (5)
C4—C5—C6—C1	1.0 (5)	C14—C22—C23—O5	−51.6 (4)
C4—C5—C6—C7	−175.9 (3)	O5—C23—C24—C25	3.3 (7)
C1—C6—C7—C9	−178.5 (3)	C22—C23—C24—C25	−178.0 (4)
C1—C6—C7—C8	0.2 (3)	C23—C24—C25—C26	−1.2 (9)
C5—C6—C7—C8	177.4 (3)	C24—C25—C26—O5	−1.3 (10)
C5—C6—C7—C9	−1.3 (5)	N3—C27—C28—C29	31.7 (3)
C9—C7—C8—N1	179.4 (3)	N3—C27—C28—C33	−149.9 (2)
C6—C7—C8—N1	0.7 (3)	C27—C28—C29—C30	178.6 (3)
C8—C7—C9—C10	98.6 (3)	C33—C28—C29—C30	0.1 (4)
C6—C7—C9—C10	−82.9 (4)	C27—C28—C33—C32	−178.1 (2)
C7—C9—C10—C11	−48.9 (3)	C29—C28—C33—C32	0.4 (4)
C7—C9—C10—N2	73.8 (3)	C28—C29—C30—C31	−0.6 (5)
C9—C10—C11—O2	−67.8 (3)	C29—C30—C31—C32	0.8 (6)
N2—C10—C11—O2	167.1 (2)	C30—C31—C32—C33	−0.3 (5)
C9—C10—C11—O1	110.3 (3)	C31—C32—C33—C28	−0.3 (4)

### Hydrogen-bond geometry (Å, °)

**Table:**

*D*—H···*A*	*D*—H	H···*A*	*D*···*A*	*D*—H···*A*
C10—H10···O3	0.98	2.36	2.751 (3)	103
C22—H22···O3	0.98	2.53	2.959 (2)	106
C27—H27B···O4	0.97	2.33	2.748 (3)	105
N1—H1···O3^i^	0.86	2.12	2.942 (3)	161
C12—H12A···Cg5^ii^	0.96	2.63	3.551 (5)	160

Symmetry codes: (i) −*x*+1, *y*−1/2, −*z*+2; (ii) *x*+1, *y*, *z*.
